# Secretome Release During In Vitro Bone Marrow-Derived Mesenchymal Stem Cell Differentiation Induced by Bio-Oss^®^ Collagen Material

**DOI:** 10.3390/ijms26083807

**Published:** 2025-04-17

**Authors:** Maria Rosa Iaquinta, Raffaella De Pace, Assia Benkhalqui, Antonio D’Agostino, Lorenzo Trevisiol, Alessia Finotti, Giulia Breveglieri, Mauro Tognon, Fernanda Martini, Elisa Mazzoni

**Affiliations:** 1 Laboratories of Cell Biology and Molecular Genetics, Section of Experimental Medicine, Department of Medical Sciences, University of Ferrara, 44121 Ferrara, Italy; mariarosa.iaquinta@unife.it (M.R.I.); mauro.tognon@unife.it (M.T.); fernanda.martini@unife.it (F.M.); 2 Center for Studies on Gender Medicine, Department of Medical Sciences, University of Ferrara, 44121 Ferrara, Italy; 3 Department of Chemical, Pharmaceutical and Agricultural Sciences, University of Ferrara, 44121 Ferrara, Italy; dpcrfl@unife.it; 4 Section of Dentistry and Maxillo-Facial Surgery, University of Verona, 37124 Verona, Italy; assia.benkhalqui@edu.unife.it (A.B.); antonio.dagostino@univr.it (A.D.); 5 Unit of Maxillo-Facial Surgery, Santa Chiara Regional Hospital, Provincial Healthcare Services Agency (APSS), 38122 Trento, Italy; lorenzo.trevisiol@univr.it; 6 Centre for Medical Sciences (CISMed), University of Trento, 38122 Trento, Italy; 7 Department of Life Sciences and Biotechnology, Section of Biochemistry and Molecular Biology, University of Ferrara, 44121 Ferrara, Italy; alessia.finotti@unife.it (A.F.); giulia.breveglieri@unife.it (G.B.); 8 Laboratory for Technologies of Advanced Therapies (LTTA), University of Ferrara, 44121 Ferrara, Italy; 9 Centre of Biotechnology, University of Ferrara, 44121 Ferrara, Italy

**Keywords:** stem cell, cytokine/chemokine, biomaterial, hydroxyapatite/collagen, bone

## Abstract

Bone diseases represent a growing healthcare challenge due to population aging and lifestyle changes. Although bone has a natural regenerative capacity, approximately 10% of fractures fail to heal properly, requiring advanced therapeutic approaches. Bone tissue engineering (BTE) has advanced the use of osteoinductive and osteoconductive biomaterials to support bone regeneration. Among them, Bio-Oss^®^ Collagen, a composite of bovine hydroxyapatite and collagen, has shown excellent biocompatibility and bioactivity properties. This study analyzes the effect of Bio-Oss^®^ Collagen on human bone marrow-derived mesenchymal stem cells (hBMSCs), assessing its osteoinductive and immunomodulatory potential. After 7 days of culture, the biomaterial modulated the expression of key genes involved in osteogenesis and chondrogenesis, which are known for their role in bone formation and maturation. At the same time, a downregulation of genes associated with bone resorption was observed. Secretome analysis revealed a controlled release of pro-regenerative cytokines, suggesting a role of the biomaterial in modulating inflammation to promote bone regeneration. Furthermore, immunofluorescence confirmed the high expression of osteocalcin and osteopontin, which are key markers of bone mineralization. These findings indicate that Bio-Oss^®^ Collagen supports osteogenesis and modulates the immune response, creating a microenvironment favorable for bone regeneration.

## 1. Introduction

The increasing prevalence of bone diseases, driven by rising life expectancy and lifestyle changes, poses a significant public health challenge. Bone has a natural ability to restore its structure and function after injury through remodeling and tissue regeneration. This process involves the coordinated actions of osteoclasts, which resorb bone, and osteoblasts, which form new bone [1]. However, approximately 10% of bone fractures fail to heal properly due to impaired bone regeneration, particularly in cases of extensive bone resections and atrophic nonunion [2,3,4]. Therefore, more effective clinical therapeutic strategies are needed. Bone tissue engineering (BTE) has been extensively studied as a potential approach for regenerating bone and cartilage fractures. BTE strategies involve the implantation of intelligent, biocompatible, osteoconductive, and osteoinductive scaffolds combined with biological cells and molecules to enhance biomaterial bioactivity, bioresorption, and tissue regeneration [1,5,6,7,8,9].

The use of biomaterials in tissue regeneration represents a promising therapeutic strategy, as these materials can guide stem cells toward differentiation and tissue remodeling. Advances in nanotechnology have significantly contributed to the development of innovative scaffolds for regenerative medicine [10,11]. These scaffolds, composed of metals, ceramics, polymers, and composites, have various clinical applications, particularly in maxillofacial, dental, and orthopedic fields [10]. Among ceramics, hydroxyapatite is considered the gold standard for bone and tooth regeneration due to its excellent biocompatibility and ability to mimic the mineral phase of bone [10,12,13]. The development of composite materials is facilitated by the versatility of polymer compounds, which allow for the combination of natural and synthetic polymers to create scaffolds suitable for both soft and hard tissue repair [1,10,14].

Bone regeneration is a complex process involving multiple stages, including inflammation, repair, and remodeling. Proper regulation of acute inflammation is essential for initiating recovery after injury [15,16]. The concept of “osteoimmunology” describes the relationship between the immune and skeletal systems, emphasizing the shared molecules—such as receptors, signaling molecules, and transcription factors—that regulate bone homeostasis and inflammation [17,18]. While inflammatory cytokines can negatively affect bone, a controlled and transient release of pro-inflammatory molecules following acute injury is crucial for tissue regeneration [17,19]. Mesenchymal stem cells (MSCs) have immunomodulatory properties that support bone repair by interacting with immune cells to influence both innate and adaptive immune responses [15,20,21]. Inflammation plays a vital role in regeneration, but excessive or prolonged inflammation can delay healing, lead to implant rejection, or cause further tissue damage [17,22]. Therefore, scaffold design should aim to promote cell proliferation, mimic natural tissue, and modulate immune responses to prevent harmful inflammation [17,22].

In our previous studies, a porous composite biomaterial composed of hydroxyapatite and collagen (Pro Osteon 200/Avitene) demonstrated excellent properties for bone regeneration in an in vitro model using human adipose-derived mesenchymal stem cells (hASCs) [8,17,23,24]. The hydroxyapatite in this biomaterial was originally sourced from coral reefs; however, due to the environmental challenges faced by these ecosystems, alternative sources are needed [17,25]. In this context, bovine bone has emerged as a promising alternative source of hydroxyapatite for hard tissue replacement in medical and dental applications [17,26].

Bio-Oss^®^ is a widely used bone substitute for bone regeneration, known not only for its biocompatibility but also for its immunomodulatory properties [17,27]. It consists of spongy bovine bone free of organic ingredients, in which the trabecular structure of the fine bone and the internal voids are preserved. Bio-Oss^®^ plays a crucial role in controlling bone regeneration and actively influences the immune environment by modulating inflammatory responses [17,28,29]. Clinical studies conducted during the last decade have reported the utility of Bio-Oss^®^ as a biocompatible material for the regeneration of intra-oral bone loss, highlighting both its osteoconductive and immunoregulatory potential [7].

A recent study highlighted the immunomodulatory effects of a biomaterial composed of bovine HA and collagen, named Bio-Oss^®^/Avitene, on maxillofacial bone regeneration by analyzing cytokine and chemokine expression in hASCs. Results show downregulation of the chemokine CCL2 and the pro-inflammatory interleukin IL-6, suggesting reduced inflammation and a favorable environment for osteointegration [17].

Human bone marrow-derived mesenchymal stem cells (hBMSCs) are essential for tissue healing and regeneration due to their self-renewal, migration, and pluripotency [8,20,30]. They play an important immunomodulatory role by secreting chemokines, cytokines, and growth factors that help repair tissues and regulate the immune response. After migrating from the bone marrow to the injury site via peripheral circulation, hBMSCs proliferate and differentiate osteogenically, supporting both tissue regeneration and immune modulation [8,30]. According to our investigations into morphology, cell biology, and epigenetics, hBMSCs make a great in vitro biological model for testing scaffolds for bone repair/regrowth and tissue engineering [8].

This study explores the immunomodulatory effects of Bio-Oss^®^ Collagen, a commercial hydroxyapatite/collagen scaffold, when seeded with hBMSCs. The primary goal of this study is to evaluate how Bio-Oss^®^ Collagen influences osteoinductivity, as well as the immune response in bone regeneration. Additionally, this work aims to better understand how Bio-Oss^®^ Collagen interacts with the inflammatory environment and its potential to regulate the expression of cytokines and chemokines in a controlled manner in hBMSCs, ultimately promoting a favorable regenerative microenvironment. By addressing these critical aspects, this study seeks to fill a gap in current research by providing new insights into the dual role of scaffolds in modulating both bone formation and immune regulation.

## 2. Results

### 2.1. Bio-Oss^®^ Collagen Modulates the Expression of Genes Involved in Skeletal and Cartilage Development in hBMSCs

**Figure 1:**
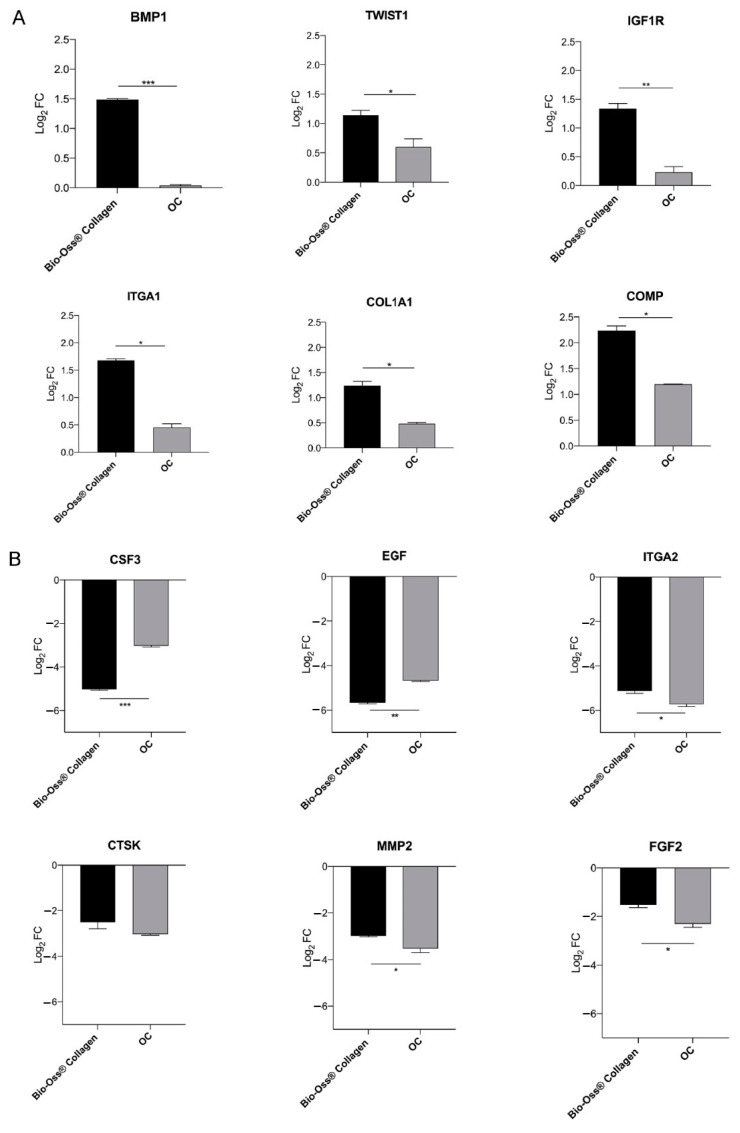
Representation of the differential osteogenic gene expression in hBMSCs cultured on Bio-Oss^®^ Collagen compared to those grown under osteogenic conditions (OCs) after 7 days. (**A**) The PCR array analysis highlights a significant upregulation of key genes involved in bone formation and extracellular matrix development. *COL1A1*, *COMP*, and *ITGA1*, fundamental components of the extracellular matrix, show a statistically significant increase in cells grown on Bio-Oss^®^ Collagen compared to OCs (* *p* < 0.05). Similarly, *BMP1* and *TWIST1*, which are essential for osteoprogenitor differentiation and bone formation, are significantly upregulated by the biomaterial (* *p* < 0.05; *** *p* < 0.001). Additionally, *IGF1R*, a receptor activated by IGF1 that enhances ALP activity and calcium deposition, is significantly upregulated in cells grown on Bio-Oss^®^ Collagen (** *p* < 0.01), confirming its role in promoting mineralization. (**B**) Conversely, genes associated with osteoclastogenesis, inflammation, and bone resorption, such as *ITGA2*, *MMP2*, *EGF*, *FGF2*, *CTSK*, and *CSF3*, are generally downregulated. Notably, *CSF3* and *EGF* show a significant reduction in cells grown on Bio-Oss^®^ Collagen compared to OCs (*** *p* < 0.001; ** *p* < 0.01), suggesting a decrease in pro-inflammatory signaling. Furthermore, *ITGA2*, *MMP2*, and *FGF2* are significantly downregulated in OCs compared to cells grown on the scaffold (* *p* < 0.05), indicating a shift in gene expression dynamics between the two conditions.

Gene expression data were normalized to TCPS control cells. The gene expression of hBMSCs grown on biomaterial was compared to hBMSCs grown in OCs. Bio-Oss^®^ Collagen induces the differential expression of several genes in hBMSCs (DEGs *n* = 12) involved in both osteogenic and chondrogenic pathways, on day 7 (Figure 1).

A total of six genes were significantly upregulated (>1 log_2_ fold change), while seven genes were significantly downregulated (<−1 log_2_ fold change) in hBMSCs grown on scaffold compared to the control cells (TCPS).

Among the upregulated genes, several play key roles in osteogenesis and chondrogenesis, including collagen type I alpha 1 (*COL1A1*), cartilage oligomeric matrix protein (*COMP*), integrin alpha 1 (*ITGA1*), proteins that constitute the extracellular matrix, bone morphogenetic protein 1 (*BMP1*), twist homolog 1 (*TWIST1*), essential for the bone formation process and osteoprogenitors differentiation and insulin-like growth factor receptor 1 (*IGF1R*), activated by a hormone called insulin-like growth factor 1 (*IGF-1*). The biomaterial significantly induced the expression of these genes compared to cells cultured in OCs (* *p* < 0.05; ** *p* < 0.01; *** *p* < 0.001) (Figure 1A, Table 1).

The six downregulated genes include integrin alpha 2 (*ITGA2*), matrix metalloproteinase 2 (*MMP2*), epidermal growth factor (*EGF*), fibroblast growth factor 2 (*FGF2*), cathepsin K (*CTSK*), and colony-stimulating factor 3 (*CSF3*). Elevated expression of these genes in bone tissue is associated with osteoclastogenesis, inflammatory cartilage degradation, and bone resorption. Notably, the biomaterial significantly downregulated *CSF3* and *EGF* compared to cells cultured in OCs (*** *p* < 0.001; ** *p* < 0.01). Additionally, *ITGA2*, *MMP2*, and *FGF2* were significantly downregulated in OCs compared to scaffold-cultured cells (* *p* < 0.05) (Figure 1B, Table 1).

### 2.2. Secretome Profile Analysis in hBMSCs: Proteins Involved in Immunomodulation

Numerous regulatory molecules, such as cytokines, chemokines, receptors, and transcription factors, link the immune and skeletal systems. In the bone marrow, bone and immune cells work together to perform various bone-related functions, including maintaining the body’s structure, regulating mineral metabolism, hematopoiesis, and bone tissue regeneration [31]. Following an acute injury, a key step in tissue regeneration is the transient and tightly regulated release of pro-inflammatory molecules, followed by a gradual decrease in inflammation as the tissue heals. Inflammation is a critical biological process that must be considered when developing biomaterial-based therapies, as it can delay wound healing or, in some cases, lead to scaffold rejection and further tissue damage [17].

**Figure 2:**
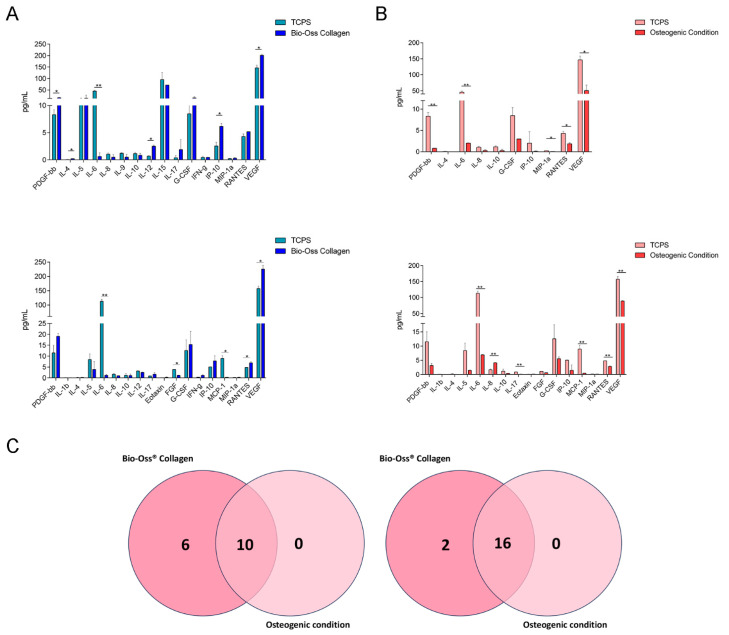
Bio-Plex analysis of cytokines/chemokines released by hBMSCs grown on Bio-Oss^®^ Collagen and in OCs. (**A**) On day 3, hBMSCs grown on Bio-Oss^®^ Collagen released 16 cytokines, with significant increases in PDGF-β, IL-4, IL-5, IL-12, G-CSF, IP-10, and VEGF compared to TCPS (* *p* < 0.05). Conversely, IL-6 was significantly overexpressed in TCPS (** *p* < 0.01). On day 7, hBMSCs on Bio-Oss^®^ Collagen released 18 cytokines, maintaining most proteins detected on day 3, except IL-9 and IL-15, which were no longer present, and IL-1β, eotaxin, FGF, and MCP-1, which newly appeared. RANTES and VEGF were significantly upregulated in scaffold-grown cells compared to TCPS (* *p* < 0.05), while IL-6, FGF, and MCP-1 were more expressed in TCPS (** *p* < 0.01). (**B**) In osteogenic conditions (OCs), hBMSCs released 10 cytokines on day 3, with IL-1β, IL-6, MIP-1, RANTES, and VEGF significantly more expressed in TCPS (* *p* < 0.05; ** *p* < 0.01). By day 7, the number of secreted cytokines increased to 16, with six new cytokines appearing (IL-1β, IL-5, IL-17, Eotaxin, FGF, and MCP-1). IL-6, IL-17, MCP-1, RANTES, and VEGF were significantly higher in TCPS (* *p* < 0.05; ** *p* < 0.01), whereas IL-8 was significantly increased in OCs (** *p* < 0.01). (**C**) The Venn diagrams compare cytokines secreted by hBMSCs in Bio-Oss^®^ Collagen and OCs. On day 3, both groups shared 10 cytokines, but the scaffold-grown cells released six additional cytokines absent in OCs. On day 7, the overlap increased to 16 cytokines, with Bio-Oss^®^ Collagen promoting the release of two additional cytokines not found in OCs. The data show that after 3 days of culture, 16 cytokines were released from cells grown on the biomaterial (Figure 2A, Table 2).

To investigate the role of the scaffold in the inflammatory and immunoregulatory processes, cytokine/chemokine release data were obtained through Bio-Plex analysis. The analysis was performed on supernatants collected from hBMSCs grown on the Geistlich Bio-Oss^®^ Collagen biomaterial, in OCs, and on plastic vessels (TCPS) after 3 and 7 days (Figure 2).

**Table 2:** List of cytokines involved in the immune response released by hBMSCs cultured on the scaffold on day 3.

	TCPS		Bio-Oss^®^ Collagen	
	Mean	SD	Mean	SD
PDGF-bb	8.320	0.905	16.095	1.850
IL-4	0.045	0.049	0.220	0.000
IL-5	10.705	3.854	14.000	14.000
IL-6	45.290	3.960	0.620	0.679
IL-8	1.050	0.198	0.490	0.490
IL-9	1.220	0.100	0.510	0.500
IL-10	1.155	0.219	0.840	0.354
IL-12	0.680	0.100	2.510	0.200
IL-15	95.130	30.745	71.450	0.100
IL-17	0.400	0.400	1.880	1.880
G-CSF	8.455	1.945	15.375	6.003
IFN-g	0.445	0.219	0.460	0.000
IP-10	2.550	0.673	6.160	0.555
MIP-1a	0.230	0.042	0.310	0.127
RANTES	4.300	0.467	5.190	0.000
VEGF	146.310	11.978	201.180	4.464

Platelet-derived growth factor subunit B (PDGF-β), interleukin 4 (IL-4), interleukin 5 (IL-5), interleukin 6 (IL-6), interleukin 8 (IL-8), interleukin 9 (IL-9), interleukin 10 (IL-10), interleukin 12 (IL-12), interleukin 15 (IL-15), interleukin 17 (IL-17), granulocyte-colony stimulating factors (G-CSFs), interferon γ (INF-γ), interferon gamma-induced protein 10 (IP-10), macrophage inflammatory protein 1α (MIP-1α), regulated on activation, normal T cell expressed and secreted (RANTES), and vascular endothelial growth factor (VEGF) were released from cells grown on the biomaterial. The most concentrated proteins in the supernatant of hBMSCs grown on the biomaterial were as follows: PDGF-β (16.095 pg/mL), IL-5 (14 pg/mL), IL-15 (71.450 pg/mL), G-CSF (15.375 pg/mL), IP-10 (6.160 pg/mL), RANTES (5.190 pg/mL), and VEGF (201.180 pg/mL). Furthermore, the protein expression of PDGF-, IL-4, IL-5, IL-12, G-CSF, IP-10, and VEGF, was significantly increased in cells grown on the biomaterial compared to TCPS. In contrast, IL-6 was significantly overexpressed in TCPS cultures compared to cells grown on the scaffold.

On day 7 of culture, the cells grown on the biomaterial released 18 cytokines (Figure 2A, Table 3). Among these were the same proteins released on day 3, with the exception of IL-9 and IL-15, which were absent on day 7.

Furthermore, among the 18 cytokines released on day 7, 4 were not present on day 3, namely, interleukin 1β (IL-1β), eotaxin, fibroblast growth factor (FGF), and monocyte chemoattractant protein-1 (MCP-1). The cytokines most abundantly secreted by hBMSCs grown on the biomaterial were PDGF-β (19.110 pg/mL), IL-5 (3.930 pg/mL), G-CSF (15.375 pg/mL), IP-10 (7.820 pg/mL), RANTES (6.930 pg/mL), and VEGF (225.280 pg/mL). The cytokines RANTES and VEGF were significantly overexpressed in hBMSCs grown on the biomaterial compared to TCPS, while IL-6, FGF, and MCP-1 were significantly overexpressed in TCPS compared to cells grown on the scaffold.

On the third day of growth, cells grown under osteogenic conditions released 10 cytokines (Figure 2B, Table 4).

Platelet-derived growth factor subunit B (PDGF-β), interleukin 4 (IL-4), interleukin 6 (IL-6), interleukin 8 (IL-8), interleukin 10 (IL-10), granulocyte-colony stimulating factors (G-CSFs), interferon gamma-induced protein 10 (IP-10), macrophage inflammatory protein 1α (MIP-1α), regulated on activation, normal T cell expressed and secreted (RANTES), and vascular endothelial growth factor (VEGF) were released from cells grown under OCs. The most concentrated in the supernatant was VEGF (50.655 pg/mL), followed by G-CSF (3 pg/mL), IL-6 (2.025 pg/mL), and RANTES (1.865 pg/mL). In particular, the cytokines IL-1β, IL-6, MIP-1, RANTES, and VEGF were released in significantly higher concentrations by TCPS compared to hBMSCs grown on the biomaterial. On day 7, there was an increase of six proteins released by cells grown under OCs (Figure 2B, Table 5).

Cytokines released on day 3 included interleukin 1β (IL-1β), interleukin 5 (IL-5), interleukin 17 (IL-17), eotaxin, fibroblast growth factors (FGFs), and monocyte chemoattractant protein-1 (MCP-1). The most concentrated cytokines in the supernatant were VEGF (88.810 pg/mL), IL-6 (6.910 pg/mL), G-CSF (5.555 pg/mL), IL-8 (4.110 pg/mL), PDGF-β (3.165 pg/mL), RANTES (2.835 pg/mL) and IL-5 (1.450 pg/mL). IL-8 was significantly overexpressed by hBMSCs in OCs compared to TCPS. Contrariwise, IL-6, IL-17, MCP-1, RANTES, and VEGF were released in significant quantities by TCPS compared to OCs. 

The Venn diagram provides insight into the evolution of cytokine secretion from day 3 to day 7 and allows for the comparison of common and distinct cytokines secreted by hBMSCs grown on the scaffold and in osteogenic conditions. On day 3 (Figure 2C, Table 6), the hBMSCs grown on the biomaterial and in OCs secreted 10 common cytokines, namely, PDGF-β, IL-4, IL-6, IL-8, IL-10, G-CSF, IP-10, MIP-1α, RANTES, and VEGF. Additionally, the cells grown on the scaffold released six cytokines, namely, IL-5, IL-9, IL-12, IL-15, IL-17, and INF-γ, which were not secreted by hBMSCs grown in OCs. On day 7 (Figure 2C, Table 6), the hBMSCs grown on the biomaterial and in OCs secreted 16 common cytokines, namely, PDGF-β, IL-1β, IL-4, IL-5, IL-6, IL-8, IL-10, IL-17, eotaxin, FGF, G-CSF, IP-10, MCP-1, MIP-1α, RANTES, and VEGF. Moreover, the cells grown on the scaffold released two cytokines, IL-12 and INF-γ, which were not secreted by hBMSCs grown in OCs.

### 2.3. Osteocalcin and Osteopontin Expression as Osteogenic Markers

To assess the potential role of the biomaterial in osteogenic induction, the expression of osteocalcin (OCN) and osteopontin (OPN) proteins was evaluated in hBMSCs.

Osteocalcin is a protein from the osteocalcin/matrix Gla-protein family, making up 1–2% of total bone protein. In humans, OCN is encoded by the BGLAP gene, is the most abundant bone matrix protein, and is primarily expressed by osteoblasts, with the ability to bind calcium ions. OCN plays a dual role in bone, regulating bone remodeling by modulating osteoblast and osteoclast activity, and acting as a regulator of bone mineralization. It is also involved in bone resorption, particularly in regulating osteoclast formation and activity [32].

Osteopontin, encoded by the SPP1 gene in humans, is a multifunctional protein essential for bone remodeling and biomineralization. OPN promotes osteoclastogenesis and osteoclastic activity through cell signaling mediated by CD44 and αvβ3. It also regulates hydroxyapatite (HAP) crystal growth and inhibits osteoblast mineralization in a phosphate-dependent manner [33].

**Figure 3:**
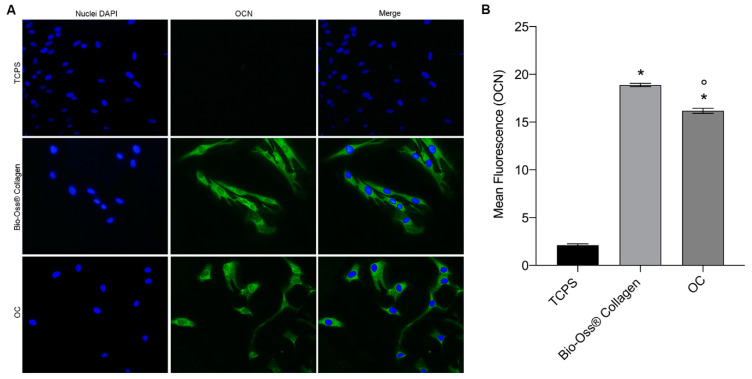
OCN protein expression in hBMSCs grown on Bio-Oss^®^ Collagen and in OCs. (**A**) The images highlight the green immunofluorescent staining of osteocalcin, demonstrating the presence of the protein at the cytoplasmic level. Cell nucleus visualization by DAPI staining (blue fluorescence). Overlap (merge) of the two images. The hBMSCs grown on the biomaterial and in OCs are positive for cytoplasmic immunolocalization of OCN; in particular, the cells grown on the scaffold show an expression of osteocalcin greater than the cells grown in OCs. Control cells show no expression of the osteocalcin protein. The slides are observed with a fluorescence microscope (TE2000E Nikon s.p.a., Florence, Italy) at a magnification of 20×. The images were obtained using ACT-1 and ACT2 software (Nikon Corporation, Tokyo, Japan) for a digital camera (DXMI200F, Nikon s.p.a., Florence, Italy). (**B**) The graph was made using GraphPad Prism 9 software and reports the average fluorescence emitted by the expression of the osteocalcin protein in stem cells grown in contact with the biomaterial, in osteogenic conditions (OCs), and in plastic vessels (TCPS). Data show significantly higher OCN expression in cells grown on the scaffold and in osteogenic conditions compared to TCPS (* *p* < 0.0001). In addition, hBMSCs grown on Bio-Oss^®^ Collagen (Geistlich Biomaterials Italia, Thiene, Italy) demonstrate a higher amount of OCN in the cytoplasm than cells grown in OCs, with a statistically significant difference (° *p* < 0.01).

To detect the immunolocalization of OCN and OPN, a fluorescent immunocytochemical analysis was performed using polyclonal antibodies for these proteins. The expression of OCN and OPN was analyzed in hBMSCs grown under three conditions, namely, (i) in contact with the biomaterial, (ii) in osteogenic conditions (OCs), and (iii) on plastic (TCPS) for 7 days. The results showed a homogeneous cytoplasmic distribution of OCN and OPN in the cells grown in both the biomaterial and osteogenic conditions. In particular, the OCN protein (Figure 3A) was more expressed in cells grown on the biomaterial than in cells grown in OCs. In contrast, TCPS showed no cytoplasmic expression of osteocalcin. This finding was further confirmed by quantifying the mean fluorescence intensity of OCN in hBMSCs, on day 7 (Figure 3B). Cells grown in contact with the biomaterial and in OCs exhibited a statistically significant increase in OCN expression compared to TCPS. Moreover, cells grown on the biomaterial showed significantly higher OCN fluorescence compared to cells in OCs.

**Figure 4:**
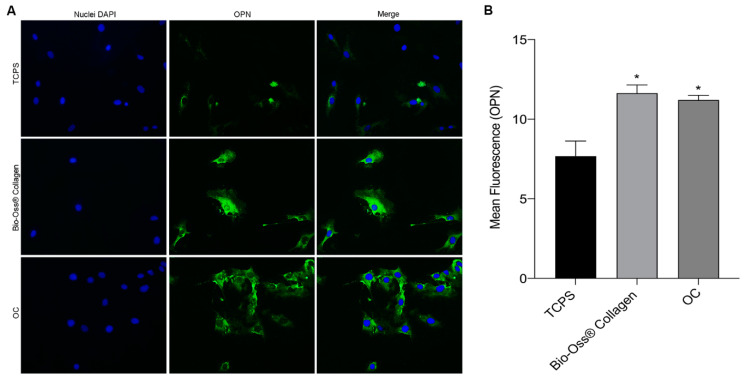
OPN protein expression in hBMSCs grown on Bio-Oss^®^ Collagen and in OCs. (**A**) The images highlight the green immunofluorescent staining of osteopontin, demonstrating the presence of the protein at the cytoplasmic level. Cell nucleus visualization by DAPI staining (blue fluorescence). Overlap (merge) of the two images. The hMSCs grown on the biomaterial and in OCs are positive for cytoplasmic immunolocalization of OPN, which is similarly expressed in cells grown on the scaffold and in OCs, without visible differences. The TCPS exhibits a slight cytoplasmatic expression of the protein. The slides were observed with a fluorescence microscope (TE2000E Nikon s.p.a., Florence, Italy) at a magnification of 20×. The images were obtained using ACT-1 and ACT2 software (Nikon Corporation, Tokyo, Japan) for a digital camera (DXMI200F, Nikon s.p.a., Florence, Italy). (**B**) The graph was made using GraphPad Prism 9 software and reports the average fluorescence emitted by the expression of the osteopontin protein in stem cells grown in contact with the biomaterial, under osteogenic conditions (OCs), and in plastic vessels (TCPS). Data show significantly higher OPN expression in cells grown on scaffolds and in osteogenic conditions compared to TCPS (* *p* < 0.05).

Meanwhile, in hBMSCs grown on the scaffold, the average fluorescence emitted by OCN was significantly higher compared to hBMSCs grown in OCs. The expression of OPN (Figure 4A) was similar in cells grown on the scaffold and in OCs, with no visible differences. TCPS showed only slight cytoplasmic expression of OPN. Quantification of the mean fluorescence confirmed the results observed in the images (Figure 4B). Both hBMSCs grown on the biomaterial and in OCs exhibited significantly higher OPN expression compared to TCPS, but no statistically significant differences were observed between the two experimental groups. These findings suggest that the biomaterial induces an osteogenic effect on the mesenchymal stem cell model, similar to the osteoinductive effects of the osteogenic medium.

## 3. Discussion

Regenerative medicine aims to restore the structural and functional integrity of damaged human organs and tissues, bringing them back to a state typical of healthy tissue. Tissue engineering, specifically, focuses on developing functionally active human tissues in vitro, which can be used clinically as biological substitutes for the repair, maintenance, and regeneration of tissues [34]. One promising approach in bone tissue engineering involves the combination of three-dimensional (3D) scaffolds, signaling molecules, and stem cells to restore the structure and functional properties of healthy bone tissue [35,36]. Numerous studies have focused on the development of new biomaterials for bone grafting and regrowth, aiming to improve essential properties such as biocompatibility, mechanical properties, osteoconductivity, osteoinductivity, healing rates, and immunomodulation [37,38]. Additionally, there is growing evidence that the innate and adaptive immune systems’ cells and molecules influence bone remodeling. The emerging field of osteoimmunology investigates these interactions, which are particularly relevant in conditions such as osteoporosis, skeletal metastases, and inflammation-induced bone loss. Several studies have shown how specific chemokines affect the differentiation and functionality of osteoclasts and/or osteoblasts. Through autocrine and paracrine signaling, skeletal cells and other cells in the bone marrow niche regulate bone production and resorption via chemokine signaling [39]. Hydroxyapatite has been used for years in bone surgery because its mineral composition and mechanical properties closely resemble those of natural bone. Collagen/hydroxyapatite composites are widely used for bone grafting, primarily due to their excellent compositional similarity to bone and their potential as drug delivery systems. Theese composite materials are currently the materials of choice for bone grafts [40]. In this study, a commercial collagen/hydroxyapatite composite biomaterial, Geistlich Bio-Oss^®^ Collagen, was analyzed for its osteoinductive and immunomodulatory biological properties in an in vitro model using hBMSCs.

Analysis of differential gene expression (DEG) showed that the biomaterial enhances the expression of genes involved in both osteogenesis and chondrogenesis, such as *COL1A1*, *COMP*, *BMP1*, *TWIST*, *ITGA1*, and *IGF1R*. Collagen is abundant in bone and skin tissues, where it forms filamentous structures that provide tensile strength. Mineralized collagen fibrils serve as the structural foundation of the bone-implant interface [41]. COL1A1, the main component of the bone extracellular matrix, plays a vital role in osteogenesis, and mutations in this gene are associated with defective bone formation [42]. The cartilage oligomeric matrix protein (COMP) is a large homopentameric glycoprotein found in the extracellular matrix of various tissues, such as cartilage, ligaments, tendons, bones (produced by osteoblasts), and synovial tissue. COMP interacts with other extracellular matrix molecules, including collagens, proteoglycans, and fibronectin. Recent studies have shown that COMP binds to members of the TGF-β protein family, including bone morphogenetic proteins (BMPs), and activates TGF-β-dependent transcriptional activity [43]. Bone morphogenetic protein 1 (BMP1), a metalloprotease, plays a critical role in osteogenesis and extracellular matrix (ECM) formation, especially in the proteolytic removal of propeptides from procollagen precursors. This process is essential for the self-assembly of mature collagen fibrils. Mutations in BMP1 lead to osteogenesis imperfecta, underscoring its significance in bone formation [44]. Twist1 is a highly homologous bHLH transcription factor that has broad expression profiles during development. Studies have shown that mice with a haploinsufficient *Twist1* gene exhibited reduced bone formation, as well as reduced proliferation and differentiation of osteoprogenitors [45]. ITGA1 forms a receptor on the cell surface for collagen and laminin when heterodimerized with the β1 subunit. Research indicates that increasing ITGA1 expression leads to the differentiation of human skeletal muscle stem cells into odontoblasts, demonstrated by the overregulation of dentin sialophosphoprotein and ALP [46]. Moreover, a recent study has shown for the first time that enhanced ITGA1 expression through receptor-ECM interaction signaling promotes osteogenic differentiation and optimizes the osteogenic potential of human periodontal ligament stem cells [47]. IGF-1R is a transmembrane receptor activated by insulin-like growth factor 1 (IGF-1). IGF-1 is a multifunctional peptide that regulates cell growth, differentiation, and the expression of extracellular matrix proteins. A recent study has demonstrated that IGF-1 and IGF-1R increase ALP activity and calcium deposition in MSCs derived from rat bone marrow in a dose-dependent manner. Additionally, IGF-1 enhances OCN expression during the later stages of osteogenesis [48].

The biomaterial also negatively modulates genes involved in bone resorption and cartilage erosion, including *CSF3*, *ITGA2*, *EGF*, *CTSK*, *MMP2*, and *FGF2*. Integrin ITGA2 is known as a collagen-binding receptor and is expressed in various tissues and cells, including fibroblasts. Studies indicate that ITGA2 plays a significant role in the inflammatory destruction of cartilage by promoting the proliferation and attachment of fibroblasts and the expression of MMPs. In arthritic conditions, deficiency in ITGA2 reduces the severity of joint pathology. Specifically, Itga2-deficient mice (Itga2−/−) display less severe clinical symptoms and significantly reduced cartilage erosion. These mice also show decreased MMP expression in both serum and fibroblast synoviocytes [48]. This protein appears to be negatively modulated by biomaterials, along with MMP2. This metalloprotease is essential for extracellular matrix remodeling and other pathological processes, including tumor progression and skeletal dysplasia. Excessive activation of MMP2 promotes osteolytic metastases, bone destruction, and osteolytic effects. MMP2 can degrade the bone matrix, facilitate osteoclastogenesis, and amplify signaling pathways that increase osteolysis in bone metastases [49].

Cathepsin K, a cysteine protease expressed by osteoclasts and synovial fibroblasts, degrades key bone and cartilage components such as type I and II collagen, osteonectin, and aggrecan. Inhibiting cathepsin K activity could help prevent bone erosion and cartilage degradation in rheumatoid arthritis. Pharmacological inhibition of cathepsin K proteolytic activity in a mouse model of rheumatoid arthritis has been shown to reduce both bone and cartilage destruction in arthritic joints [34]. FGF2 has been found to partially inhibit the mineralization of BMSCs by regulating gene expression of CREB and the RANKL, involved in osteoclastogenesis. Studies show that BMSCs deficient in FGF2 exhibit increased mineralization capacity and decreased osteoclastogenic gene expression. Consequently, FGF2-knockout mice display increased bone mass and reduced expression of osteoclast-related markers, which is linked to moderate inhibition of ERK signaling [48]. CSF3 is a glycoprotein that promotes the proliferation of osteoclastic progenitor cells. Previous studies have demonstrated that CSF3 release in bone tissue disrupts the balance between bone formation and resorption, leading to pathological conditions associated with excessive bone resorption, such as periodontitis and osteoporosis [50].

The osteogenic potential of the Geistlich Bio-Oss^®^ biomaterial has also been demonstrated through immunofluorescence analysis of two key osteogenic marker proteins, namely, osteopontin and osteocalcin. These proteins are highly expressed in the cytoplasm of hBMSCs grown on the biomaterial, in contrast to the TCPS. Osteocalcin and osteopontin are essential in regulating bone mineralization and cell adhesion, playing significant roles in the formation and maintenance of the bone matrix. Osteocalcin is a non-collagenous protein primarily produced by osteoblasts and constitutes about 10–20% of non-collagenous proteins in the extracellular bone matrix. It occurs in the late stages of osteoblast maturation, increasing notably during bone matrix mineralization. OCN has a strong affinity for hydroxyapatite, the main mineral component of bone, which helps stabilize the bone matrix and regulate the growth of hydroxyapatite crystals, influencing bone density and strength [51]. Osteopontin is another non-collagenous protein of the bone matrix. It is produced by osteoblasts, osteoclasts, and chondrocytes. Osteopontin plays a crucial role in osteogenesis by promoting osteoblast migration and differentiation. Additionally, osteopontin regulates bone mineralization by hydroxyapatite crystal deposition and contributes to bone remodeling [52]. The combined action of osteocalcin and osteopontin is essential for maintaining a proper balance between bone formation and resorption, ensuring the health and functionality of bone tissue.

Various regulatory molecules, such as cytokines, chemokines, receptors, and transcription factors, act as intermediaries between the immune and skeletal systems. Within the bone marrow, interactions between bone cells and immune cells are essential for various bone-related functions, including providing structural support, regulating mineral metabolism, producing blood cells, and regenerating bone tissue [53]. Following a bone injury, a crucial step in the regeneration process is the controlled and temporary release of pro-inflammatory mediators, followed by a gradual reduction in inflammation as tissue repair progresses. Inflammation is a vital biological mechanism to consider when designing biomaterials for medical applications, as it plays a key role in initiating the healing process in its early stages. However, if inflammation persists, it can prolong the healing process or, in some cases, result in scaffold rejection and further tissue damage [54].

To assess the role of the scaffold in modulating inflammatory and immune responses in hBMSC cultures compared to TCPS, cytokine and chemokine release was analyzed using the Bio-Plex system. On day 3 of culture, hBMSCs grown on the biomaterial released several cytokines, including the most abundant ones, namely, PDGF-β, IL-15, G-CSF, IP-10, RANTES, and VEGF. Notably, PDGF-bb, IP-10, VEGF, and G-CSF were released from hBMSCs cultured on the material in significantly higher amounts than in control cells. Additionally, IL-4 and IL-12 were also detected. PDGF-β is known to positively influence bone regeneration by stimulating osteogenesis through osteoblast activation and promoting the synthesis of the extracellular matrix. Furthermore, PDGF-β modulates the inflammatory response, that is, while it promotes an acute inflammatory reaction, it also favors the transition to resolution, preventing chronic inflammation. This dual role helps reduce local inflammation and supports tissue healing [55]. IL-4 is recognized for its ability to induce macrophage polarization toward the M2 phenotype, which is anti-inflammatory and aids tissue repair. In osteogenic contexts, IL-4 has been shown to promote osteoblast differentiation, enhance bone mineralization, and contribute to osteogenesis. Its anti-inflammatory effects are crucial to preventing the persistence of chronic inflammation, which could hinder bone regeneration [56].

The impact of IL-15 on the immune system and inflammation may indirectly influence bone regeneration. The controlled release of IL-15 during the initial inflammatory phase could stimulate the production of other cytokines that promote healing, such as VEGF. It enhances nutrient supply and the influx of osteoprogenitor cells to the injury site by encouraging blood vessel formation [57].

IL-12 is a cytokine primarily produced by dendritic cells and macrophages; it plays a role in promoting both innate and adaptive immune responses. IL-12 also has potential effects on immune responses at the tissue regeneration level. Some studies suggest that IL-12 may modulate osteogenesis by enhancing macrophage function and the production of bone-promoting cytokines, particularly in a controlled inflammatory environment [58].

VEGF, G-CSF, IP-10, and RANTES are cytokines generally associated with new blood vessel formation, immune cell migration, and bone remodeling—all essential processes for bone tissue regeneration after injury. Although G-CSF is more commonly recognized for its role in neutrophil activation, it also directly affects osteogenesis. Several studies have shown that G-CSF can stimulate osteoblast proliferation, the cells responsible for new bone formation, and promote the differentiation of mesenchymal stem cells into osteoblasts. Additionally, G-CSF modulates inflammation and promotes bone cell production, accelerating fracture healing [59]. VEGF is crucial for vascular regeneration, providing the necessary nutritional support for new bone tissue growth. The high concentrations of VEGF found in the supernatant of hBMSCs cultured on the biomaterial suggest that the material might positively influence neovascularization, an essential step in regenerating damaged bone tissue [60]. IP-10 and RANTES are pro-inflammatory cytokines, and their controlled release is vital for recruiting immune cells to initiate the healing process. The increased expression of RANTES and VEGF by the material may indicate new blood vessel formation, activation of inflammatory responses, and stimulation of immune cell chemotaxis, all of which are beneficial for tissue growth and recovery [61].

In contrast to the cytokines described above, IL-6 is significantly overexpressed in the control (TCPS) compared to cells grown on the biomaterial. IL-6 is a key cytokine in the acute inflammatory response, but excessive levels of this molecule can delay healing and increase the risk of scaffold rejection. This suggests that fine regulation of the inflammatory response by the biomaterial may be a critical factor for the success of bone regeneration [62].

On day 7, the cytokines predominantly secreted by hBMSCs grown on the biomaterial are PDGF-β, IL-5, G-CSF, IP-10, RANTES, and VEGF, with the latter two being expressed at significantly higher levels than in TCPS. As on day 3, IL-6 is significantly released from TCPS compared to cells grown on the biomaterial, with two new cytokines—FGF and MCP-1—not present on day 3. FGF promotes the proliferation of various cell types, including fibroblasts and mesenchymal cells. However, excessive FGF release can increase the fibroblast population at the expense of osteoblasts, thereby hindering bone formation. This imbalance could compromise proper bone matrix formation and delay mineralization [63]. MCP-1 is a chemokine that regulates the recruitment of monocytes and immune cells to the site of inflammation. While its role in tissue repair and inflammation modulation is important, its overexpression can negatively impact osteogenesis. High MCP-1 concentrations may prolong inflammation, delaying the transition from the inflammatory phase to bone regeneration. Additionally, MCP-1 has been shown to inhibit the differentiation of osteoprogenitors into osteoblasts, reducing the ability to form new bone. Excessive MCP-1 activation can decrease direct osteogenesis and increase bone resorption [64].

When comparing the cytokine profile of cells grown under osteogenic conditions to TCPS, a significant release of PDGF-bb, IL-6, MIP-1α, RANTES, and VEGF is observed in the control group on day 3, compared to hBMSCs grown under osteogenic conditions. On day 7, there is an increase in the number of cytokines released by cells cultured under osteogenic conditions, including IL-1a, IL-5, IL-17, Eotaxin, FGF, and MCP-1. Among these, IL-6, IL-17, MCP-1, RANTES, and VEGF are released in significantly higher amounts by control cells compared to those grown under osteogenic conditions. In contrast, IL-8 is significantly overexpressed in hBMSCs cultured in osteogenic conditions compared to control cells. Recent studies have shown that IL-8 can stimulate osteoprogenitor proliferation and differentiation into osteoblasts. This effect is primarily mediated by the interaction between IL-8 and its receptors (CXCR1 and CXCR2) on mesenchymal cells. Activation of these receptors by IL-8 leads to osteoprogenitor differentiation into osteoblasts, thereby promoting increased bone mineralization [65].

The comparison of the cytokine profiles between hBMSCs grown on the biomaterial and control cells (TCPS) reveals that cells grown on the biomaterial exhibit a cytokine profile conducive to bone regeneration, largely through the modulation of inflammatory and angiogenic responses. In contrast, control cells tend to express inflammatory cytokines more prominently, indicating a stronger inflammatory response. The release of cytokines such as PDGF-β, VEGF, IL-4, G-CSF, and RANTES suggests an environment favorable to neoangiogenesis, cell chemotaxis, and the promotion of osteoblast differentiation. Additionally, the reduced production of inflammatory cytokines like IL-6 implies that the biomaterial helps modulate the inflammatory response in a way that avoids detrimental effects on healing. Conversely, control cells show a more pronounced inflammatory profile, particularly with excess IL-6, suggesting that, without the biomaterial’s support, cells may express stronger inflammation.

The comparison between the cytokine profiles of hBMSCs grown under osteogenic conditions (OCs) and control cells (TCPS) reveals significant differences in cytokine secretion, suggesting that the osteogenic environment influences both the inflammatory response and the production of bioactive factors. Notably, the high production of inflammatory cytokines like IL-6 in control cells may indicate that the absence of osteogenic stimuli leads to an accumulation of inflammatory signals. An interesting observation is the significantly higher expression of IL-8 in hBMSCs grown under osteogenic conditions compared to control cells. IL-8 is a cytokine involved in neutrophil chemotaxis and the inflammatory response, but its overexpression in osteogenic conditions may also reflect a tissue remodeling process specific to osteogenic differentiation. This finding suggests that, even within an osteogenic context, hBMSCs continue to respond to inflammatory signals, but in a targeted manner, directing the inflammatory response to support the healing and bone differentiation process.

The analysis of the results obtained through the Venn diagram clearly illustrates how the cytokine secretion of hBMSCs grown on the biomaterial and under osteogenic conditions evolves over time, with significant overlap in the cytokines released.

On day 3, both hBMSCs grown on the biomaterial and those under osteogenic conditions secrete 10 common cytokines involved in key processes for bone regeneration and tissue repair, including inflammation modulation, promotion of neoangiogenesis, cell migration, and osteoblast proliferation. For instance, the presence of PDGF-β, known for its role in stimulating osteogenesis and promoting bone healing, suggests that the biomaterial may support the activation of regenerative processes in a manner similar to osteogenic conditions. Additionally, VEGF, which is critical for blood vessel formation, is present in both groups, indicating that the biomaterial may promote vascularization, which is essential for regenerating damaged bone tissue.

On day 7, there is an increase in the number of common cytokines, with 16 cytokines released. In particular, the sustained secretion of PDGF-β and VEGF, which support osteogenesis and neoangiogenesis, suggests that the biomaterial may stimulate both bone growth and the formation of new blood vessels. Moreover, the activation of IL-4, known for its anti-inflammatory effects, indicates that the biomaterial is also contributing to the modulation of the inflammatory response.

In conclusion, this study explores the potential role of Geistlich Bio-Oss^®^ Collagen in promoting bone regeneration through its influence on osteogenic and immunomodulatory processes. The biomaterial appears to enhance the gene and protein expression of key osteogenic markers, while also downregulating genes associated with bone resorption and inflammation. Additionally, its potential to modulate the inflammatory response by promoting pro-regenerative cytokines such as PDGF-β, VEGF, and G-CSF, while reducing elevated levels of IL-6, suggests that it may help create a more balanced environment for bone healing.

## 4. Materials and Methods

### 4.1. Experimental Design

The immunomodulatory properties and osteoinductive potential of the composite commercial scaffold, Bio-Oss^®^ Collagen (Geistlich Biomaterials Italia, Thiene, Italy), were analyzed in human hBMSCs seeded on biomaterial. These properties were compared with hBMSCs cultured in osteogenic conditions (OCs) (positive control) and those grown in a monolayer on tissue culture polystyrene (TCPS) in the basal medium (negative control).

The osteoinductive potential of the scaffold was assessed by evaluating the gene expression (mRNA) of key osteogenesis-related genes in hBMSCs using a real-time PCR array. Additionally, immunocytochemical analysis was performed to detect two crucial osteogenic proteins—osteopontin and osteocalcin—after 7 days of culture. The choice of the 7-day time point for hBMSC analysis is based on evidence that, within this period, both early and late markers of osteogenic differentiation emerge [66]. Additionally, the effect of the scaffold on the inflammatory response of hBMSCs was assessed by quantifying major cytokines and chemokines involved in pro-inflammatory and anti-inflammatory responses using the Bioplex technique, after 3 and 7 days of culture. The choice of the 3- and 7-day time points for immunomodulation analysis aims to investigate whether the inflammatory process follows a pattern characterized by an initial peak followed by an anti-inflammatory response. This would mimic the in vivo dynamics observed at the bone injury site during the healing process [67].

### 4.2. Human BMSC Culture 

hBMSCs were obtained from Lonza, Milan, Italy (PT-2501) as cryopreserved cells at passage one. Characterization by flow cytometry analysis (FCA) confirmed the expression of positive MSC markers (CD29, CD73, and CD90) and the absence of hematopoietic markers (CD14 and CD45). Cells were expanded in α-minimum essential medium (α-MEM) (Lonza, Milan, Italy) supplemented with 20% fetal bovine serum (FBS) and 2% antibiotics (Pen/Strep 10,000 U/mL) at a density of 5000 cells/cm^2^ in T75 flasks at 37 °C with 5% CO_2_ in a humidified atmosphere.

At passage two, hBMSCs were assigned to three experimental groups:

(i) hBMSCs seeded onto block-shaped Bio-Oss^®^ Collagen (100 mg, approx. 5.0 mm × 5.0 mm × 7.0 mm) in 1 mL of α-MEM supplemented with 20% FBS and 2% Pen/Strep, in 24-well plates;
(ii) OC group (positive control): hBMSCs were cultured in 1 mL of differentiation Bullekit^TM^ osteogenic medium (Lonza, Milan, Italy), which contains osteogenic basal medium (Lonza, Milan, Italy) and osteogenic SigleQuotes^TM^ (dexamethasone, ascorbate, mesenchymal cell growth supplement, L-glutamine, and β-glycerophosphate) (Lonza, Milan, Italy), in 24-well plates;
(iii) TCPS (negative control): hBMSCs were grown as a monolayer in standard culture plates using basal medium α-MEM, supplemented with 20% FBS and 2% Pen/Strep, in 24-well plates.

hBMSCs were seeded on the biomaterial with 200 μL of cell suspension containing 1 × 10^4^ cells [33]. The cell suspension was subjected to shaking every 15 min in order to maximize cell-scaffold interaction. After 2 h, α-MEM medium was added to reach a final volume of 1 mL. In TCPS and OCs, hBMSCs were seeded on a well plate with a cell suspension containing 1 mL of 5 × 10^3^ cells in α-MEM and osteogenic medium, respectively. Cells on the biomaterial were seeded in a higher number compared to the monolayer cells on the plastic well plate, considering the larger surface area provided by the biomaterials. Cultures were maintained at 37 °C and 5% CO_2_, and the medium was replaced every 2 days until analyses [24,68,69,70].

### 4.3. Bio-Oss^®^ Collagen Material

Geistlich Bio-Oss^®^ Collagen is a composite biomaterial made up of 90% granules of Geistlich Bio-Oss^®^ and 10% collagen of porcine origin. Cells were seeded onto a block-shaped biomaterial (100 mg, approx. 5.0mm × 5.0mm × 7.0mm), providing a 3D environment that mimics physiological conditions. The particles of Geistlich Bio-Oss^®^ guarantee Geistlich Bio-Oss^®^ Collagen all the advantages of the scientifically recognized biomaterial as the leading product in the field of regenerative dentistry. The 10% collagen makes it moldable and easy to handle. The regenerative potential of Geistlich Bio-Oss^®^ Collagen clearly distinguishes it from simple collagen sponges. Geistlich Bio-Oss^®^ Collagen is used for the most diverse indications, including ridge preservation, minor bone increases, and periodontal regeneration. Collagen is reabsorbed after a few weeks and does not replace the barrier function of a membrane.

### 4.4. RNA Isolation, cDNA Synthesis, and RT^2^ Profiler^™^ PCR Array Analyses of Human Osteogenesis Genes

The RT^2^ profiler PCR array amplification test analyzed the expression of genes involved in osteogenesis (mRNA). RNA was extracted from the three following experimental groups after 7 days of culture: stem cell cultures grown in contact with plastic (TCPS), stem cell cultures grown under osteogenic soil conditions (OCs), and stem cell cultures grown on biomaterial. Total RNA was extracted through RNeasy Plus Micro Kit (Qiagen, Milan, Italy). The RNA sample was purified from genomic DNA with buffer GE for 5 min at 42 °C. RNA quality and quantity were assessed using a Nanodrop spectrophotometer (ND-1000, NanoDrop Technologies, Wilmington, DE, USA) and stored at −80 °C until the time of the analysis [8,24,71]. Purified RNA from hBMSCs was reverse-transcribed into cDNA using the RT^2^ First Strand cDNA Kit (catalog no. 330404, Qiagen, Milan, Italy). The Human Osteogenesis RT^2^ Profiler PCR array (catalog no. PAHS-026Z, Qiagen, Milan, Italy) utilized specific primer sets to assess the expression of genes involved in various pathways, including osteogenic differentiation, cartilage condensation, ossification, bone metabolism, mineralization, calcium binding and homeostasis, extracellular matrix protease inhibition, adhesion molecules, cell-to-cell adhesion, extracellular matrix interactions, and growth factors [24]. RT-PCR was performed using the SYBR Green method on a CFX96 Touch PCR detection system (Bio-Rad, Milan, Italy). The PCR reaction protocol included an initial denaturation step at 95 °C for 10 min, followed by 40 cycles of denaturation at 95 °C for 15 s, with annealing and extension at 60 °C for one minute.

For data analysis, the ΔCt values of each gene were calculated for each experimental group. The housekeeping gene *RPLP0* (Ribosomal Protein, Large, P0) was used to normalize results, as its expression remained stable across all experimental groups. Gene expression changes were analyzed using the 2^−ΔΔCt^ method. Statistical analysis was performed by comparing the ΔCt values of modulated genes in hBMSCs cultured on biomaterials and under osteogenic conditions with those in TCPS. All reactions were conducted in triplicate.

### 4.5. Bio-Plex Pro Human Cytokine 27-Plex Assay

BMSC-derived supernatant samples were collected on different days (days 3 and 7). Cytokines, chemokines, and growth factors were measured by Bio-Plex Pro Human Cytokine 27-plex Assay (no. M50-0KCAF0Y, Bio-Rad Laboratories, Hercules, CA, USA). Concentrations of IL-1b, IL-1RA, IL-2, IL-4, IL-5, IL-6, IL-7, IL-8, IL-9, IL-10, IL-12 (p70), IL-13, IL-15, IL-17, eotaxin, FGF-basic, G-CSF, GM-CSF, IFN-g, IP-10, MCP-1, MIP-1a, MIP-1b, RANTES, PDGF, VEGF, and TNF-a were simultaneously evaluated using multiplex bead-based sandwich immunoassay kits. Assays were performed following the manufacturer’s instructions. A total of 27 distinct sets of fluorescently dyed beads, each loaded with capture monoclonal antibodies specific to the cytokines being tested, were used for the analysis. A 50 μL aliquot of pre-diluted beads (1X) was added to the wells of a 96-well plate. After washing with Bio-Plex Wash Buffer, 50 μL of samples and standards were incubated in the plate for 30 min. Following incubation and washing, 25 μL of pre-diluted biotinylated antibody (1X) was added for an additional 30 min, followed by another washing step. Next, 50 μL of pre-diluted streptavidin-PE (1X) fluorophore-conjugated reagent was introduced into the wells and incubated for 10 min, followed by another washing step. Finally, beads were resuspended in 125 μL of assay buffer for 30 s. Samples were analyzed using a Bio-Rad 96-well plate reader with the Bio-Plex Suspension Array System and Bio-Plex Manager software (Version 3.0, Bio-Rad Laboratories, Hercules, CA, USA).

High standard curves were established for each soluble factor, ranging from 1.95 to 32,000 pg/mL, with a minimum detectable dose of <10 pg/mL. The formation of distinct sandwich immune complexes on different bead sets was measured and quantified using the Bio-Plex system. A 50 μL sample was taken from each well, and the fluorescent signal from at least 100 beads per region (chemokine/cytokine) was recorded. Values with a coefficient of variation exceeding 10% were excluded from the final analysis. All data were normalized to the total protein content of each secretion sample, with protein recovery determined using the bicinchoninic acid (BCA) method [72,73]. All reactions were conducted in triplicate.

### 4.6. Immunofluorescence of the Osteogenic Markers—Osteopontin and Osteocalcin

The expression of osteocalcin and osteopontin proteins was assessed by immunofluorescence in hBMSC, after 7 days of culture in the three different experimental conditions. Initially, cells were washed with PBS-1X and fixed with a 1:1 methanol–acetone solution for seven minutes at −20 °C. Cells were then treated with 0.1% Triton X-100 for 10 min, washed with PBS-1X, and incubated with primary antibodies specific for osteocalcin (catalog no. PA1-32149, Thermo Fisher Scientific, Rockford, IL, USA) and osteopontin (catalog no. PA5-11849, Thermo Fisher Scientific, Rockford, IL, USA), both diluted 1:50 in PBS-1X, for one hour at 37 °C.

Following incubation, cells were washed three times with PBS-1X for five minutes each before being incubated with the secondary Alexa Fluor 488-conjugated anti-rabbit antibody (catalog no. A-11008, Thermo Fisher Scientific, Milan, Italy), diluted 1:2 in PBS-1X + 0.1% BSA. After three additional five-minute washes with PBS-1X, the slides were mounted with DAPI (4′,6-diamidino-2-phenylindole, 0.5 μg/mL) for nuclear staining and observed under a fluorescence microscope (TE2000E, Nikon S.p.A., Florence, Italy). Image processing was performed using ACT-1 and ACT-2 software (Nikon Corporation, Tokyo, Japan) for the DXM1200F digital camera (Nikon S.p.A., Florence, Italy).

### 4.7. Data Analysis and Statistics

Statistical analyses of experiments, performed in triplicate, were carried out by using Prism 9 (GraphPad Software, La Jolla, CA, USA); A *p*-value < 0.05 was considered significant. For real-time PCR data analysis, values were normalized to housekeeping gene RPLP0, and the gene expression was analyzed by the 2^−ΔΔCt^ method. The Log2 FC < −1 or >+1 was considered significant. Statistical analysis was performed comparing the ΔCt values of hBMSCs grown on the scaffold and in OCs with the ΔCt values of TCPS [8,24]. Subsequently, the ΔCt values of hBMSCs cultured on the scaffold were compared to those of OCs using *t*-test. For the Bio-Plex assay, data were analyzed using the Bio-Plex Manager software version 3.0 (Bio-Rad Laboratories, Hercules, CA, USA). Standard levels between 80 and 120% of the expected values were considered to be accurate and were used. In general, at least 5 standards were accepted and used to establish standard curves following a 5-parameter logistic regression model (5PL). Sample concentrations were immediately interpolated from the standard curves. Values were expressed as pg/mL and presented as the mean SD. Statistical analysis was performed using an ANOVA test, comparing protein expression levels in cells grown on the scaffold and in OCs with protein expression levels of TCPS [72,73].

## 5. Conclusions

This study explores the potential role of Bio-Oss^®^ Collagen in promoting bone regeneration by influencing osteogenic and immunomodulatory processes. The biomaterial appears to enhance the expression of key osteogenic genes, such as *COL1A1*, *BMP1*, and *IGF1R*, which are involved in extracellular matrix formation, collagen maturation, osteoblast differentiation, and osteogenic signaling. Additionally, Bio-Oss^®^ Collagen seems to downregulate genes associated with bone resorption and inflammation, including *MMP2* and *CSF3*, which are linked to extracellular matrix degradation and osteoclast activation.

Furthermore, the biomaterial seems to support the expression of osteocalcin and osteopontin, non-collagenous proteins involved in bone mineralization and remodeling. The increased presence of these markers in hBMSCs cultured with Bio-Oss^®^ Collagen suggests its potential osteoinductive properties, implying that it may help mimic the natural bone remodeling environment.

Bio-Oss^®^ Collagen promotes the secretion of PDGF-β, VEGF, and G-CSF, which are known to play a role in angiogenesis, cell recruitment, and tissue remodeling. Moreover, the observed reduction in IL-6, a pro-inflammatory cytokine, suggests that Bio-Oss^®^ Collagen may contribute to balancing the inflammatory response, although further studies are needed to fully understand its impact on immune modulation.

Given its widespread clinical use, Bio-Oss^®^ Collagen has already shown effectiveness in various orthopedic, dental, and maxillofacial applications. Our findings offer additional molecular insights into its potential mechanism of action. Further research on its modulation of osteogenesis and immune responses could help refine its clinical applications.

## Figures and Tables

**Table 1:** Fold change (FC) values of deregulated genes in hBMSCs grown on Bio-Oss^®^ Collagen and under OCs on day 7.

**Upregulated Genes Bio-Oss^®^ Collagen**			**Upregulated Genes OCs**		
**No.**	**Symbol**	**Fold Change (FC)**	**No.**	**Symbol**	**Fold Change (FC)**
1	COMP	2.17	1	COMP	1.19
2	ITGA1	1.66	2	ITGA1	0.41
3	BMP1	1.48	3	BMP1	0.02
4	IGF1R	1.28	4	IGF1R	0.16
5	COL1A1	1.18	5	COL1A1	0.46
6	TWIST1	1.08	6	TWIST1	0.51
**Downregulated genes Bio-Oss^®^ Collagen**			**Downregulated genes OCs**		
**No.**	**Symbol**	**Fold change (FC)**	**No.**	**Symbol**	**Fold change (FC)**
1	EGF	−5.64	1	EGF	−4.64
2	CSF3	−5.06	2	CSF3	−3.06
3	ITGA2	−5.06	3	ITGA2	−5.64
4	MMP2	−2.94	4	MMP2	−3.64
5	CTSK	−2.32	5	CTSK	−3.06
6	FGF2	−1.43	6	FGF2	−2.39

**Table 3:** List of cytokines involved in the immune response released by hBMSCs cultured on the scaffold on day 7.

	TCPS		Bio-Oss^®^ Collagen	
	Mean	SD	Mean	SD
PDGF-bb	11.515	3.500	19.110	1.414
IL-1b	0.080	0.000	0.055	0.049
IL-4	0.130	0.170	0.220	0.000
IL-5	8.405	2.609	3.930	3.564
IL-6	113.235	6.682	1.195	0.403
IL-8	1.710	0.255	0.970	0.000
IL-10	1.185	0.700	1.080	0.693
IL-12	3.190	0.100	2.510	0.200
IL-17	0.860	0.000	1.670	0.877
Eotaxin	0.075	0.064	0.290	0.198
FGF	3.900	0.100	1.150	0.200
G-CSF	12.585	4.914	15.375	6.003
IFN-g	0.370	0.000	1.180	0.339
IP-10	5.040	0.000	7.820	2.348
MCP-1	8.930	1.329	0.290	0.100
MIP-1a	0.180	0.100	0.220	0.200
RANTES	4.820	0.000	6.930	0.481
VEGF	157.330	8.895	225.280	13.630

**Table 4:** List of cytokines involved in the immune response released by hBMSCs grown under osteogenic conditions on day 3.

	TCPS		Osteogenic Condition	
	Mean	SD	Mean	SD
PDGF-bb	8.320	0.905	0.810	0.000
IL-4	0.045	0.049	0.010	0.000
IL-6	45.290	3.960	2.025	0.064
IL-8	1.050	0.198	0.275	0.233
IL-10	1.155	0.219	0.265	0.318
G-CSF	8.455	1.945	3.000	0.000
IP-10	2.030	2.673	0.100	0.100
MIP-1a	0.230	0.042	0.040	0.000
RANTES	4.300	0.467	1.865	0.233
VEGF	146.310	11.978	50.655	17.260

**Table 5:** List of cytokines involved in the immune response released by hBMSCs grown under osteogenic conditions on day 7.

	TCPS		Osteogenic Conditions	
	Mean	SD	Mean	SD
PDGF-bb	11.515	3.500	3.165	0.658
IL-1b	0.080	0.000	0.025	0.021
IL-4	0.130	0.170	0.010	0.000
IL-5	8.405	2.609	1.450	0.100
IL-6	113.235	6.682	6.910	0.127
IL-8	1.710	0.255	4.110	0.127
IL-10	1.185	0.700	0.435	0.078
IL-17	0.860	0.100	0.080	0.100
Eotaxin	0.075	0.064	0.150	0.000
FGF	1.150	0.000	0.640	0.000
G-CSF	12.585	4.914	5.555	0.700
IP-10	5.040	0.000	1.450	1.909
MCP-1	8.930	1.329	0.470	0.000
MIP-1a	0.180	0.000	0.140	0.057
RANTES	4.820	0.000	2.835	0.219
VEGF	157.330	8.895	88.810	1.966

**Table 6:** List of common and uncommon cytokines of cytokines/chemokines released by hBMSCs grown in contact with the scaffold and under osteogenic conditions (OCs) on days 3 and 7.

Common Cytokines Secreted on Day 3:		Common Cytokines Secreted on Day 7:	
Bio-Oss^®^ Collagen	Osteogenic Conditions	Bio-Oss^®^ Collagen	Osteogenic Conditions
PDGF-β	PDGF-β	PDGF-β	PDGF-β
IL-4	IL-4	IL-1β	IL-1β
IL-6	IL-6	IL-4	IL-4
IL-8	IL-8	IL-5	IL-5
IL-10	IL-10	IL-6	IL-6
G-CSF	G-CSF	IL-8	IL-8
IP-10	IP-10	IL-10	IL-10
MIP-1α	MIP-1α	IL-17	IL-17
RANTES	RANTES	Eotaxin	Eotaxin
VEGF	VEGF	FGF	FGF
Uncommon cytokines secreted on day 3:		G-CSF	G-CSF
IL-5		IP-10	IP-10
IL-9		MCP1	MCP1
IL-12		MIP-1α	MIP-1α
IL-15		RANTES	RANTES
IL-17		VEGF	VEGF
INF-γ		Uncommon cytokines secreted on day 7:	
		IL-12	
		INF-γ	

## Data Availability

Datasets are available upon reasonable request to the corresponding author.
